# Clinical indicators of acute deterioration in persons who reside in residential aged care facilities: A rapid review

**DOI:** 10.1111/jnu.12819

**Published:** 2022-10-20

**Authors:** Shirley Chambers, Amy Spooner, Christina Parker, Leanne Jack, Linda Schnitker, Elizabeth Beattie, Patsy Yates, Margaret MacAndrew

**Affiliations:** ^1^ School of Nursing Queensland University of Technology Brisbane Queensland Australia; ^2^ School of Nursing, Midwifery and Social Sciences Central Queensland University Brisbane Queensland Australia; ^3^ Bolton Clarke Kelvin Grove Queensland Australia; ^4^ Dementia Collaborative Research Centre Queensland University of Technology Brisbane Queensland Australia; ^5^ Faculty of Health, Office of the Executive Dean Queensland University of Technology Brisbane Queensland Australia

**Keywords:** aged specific care, clinical assessment tools, clinical deterioration, clinical indicator, early warning score, rapid review, residential care

## Abstract

**Purpose:**

To identify the clinical indicators of acute deterioration in residents and the factors that influence residential aged care facility staff's identification of these.

**Design:**

Rapid review and narrative synthesis.

**Methods:**

The WHO and Cochrane Rapid Review Methods Group recommendations guided the review processes. CINAHL, Medline, PubMed, and the Cochrane Library were searched from 2000 to January 2022. Data related to clinical indicators of deterioration were categorized using the Airway, Breathing, Circulation, Disability, Exposure assessment framework, and factors influencing detection were grouped as consumer (resident and family), aged care workforce, and organization factors.

**Results:**

Twenty publications were included of which 14 informed clinical indicators; nine highlighted factors that influence staff's identification of these and three informed both. Included article were collectively below moderate quality. Most clinical indicators were grouped into the ‘Disability’ category with altered level of consciousness, behavior, and pain identified most frequently. Few studies reported more traditional indicators of deterioration used in the general population – changes in vital signs. The most common factors influencing the detection of acute deterioration were organizational and workforce‐related including resource, knowledge, and confidence deficits.

**Conclusion:**

Findings suggest subtle changes in resident's health status, rather than focusing primarily on physiologic parameters used in early warning tools for acute care settings, should be recognized and considered in the design of early warning tools for residential aged care facilities.

**Clinical Relevance:**

Early warning tools sensitive to the unique needs of residents and support for aged care facility staff are recommended to improve the capacity of aged care facility care staff to identify and manage acute deterioration early to avoid hospitalization.

## INTRODUCTION

Persons who live in residential aged care facilities (residents) are often frail and have complex physical, cognitive and social care needs, multiple morbidities, high‐symptom burden, and reduced quality of life (Eagar et al., 2020). In Australia, these residents have a greater likelihood of being transferred to emergency departments and hospitalized compared to their non‐residential aged care residing counterparts (Australian Institute of Health and Welfare, 2019). Individuals aged >50 years not living in residential aged care facilities, in 2016–17, had fewer emergency department presentations (14%) and fewer hospital admissions (31%) than their residential aged care facilities‐residing counterparts (almost 32% and 37%, respectively) (Australian Institute of Health and Welfare, 2019). However, there is evidence that around 13–40% of hospital admissions involving residents could be avoided (Laging et al., 2018) and that a transfer of residents to an emergency department and/or hospital admission place them at higher risk of developing delirium and other hospital‐acquired adverse events (Fox et al., 2020). The early detection of acute deterioration, a sudden change in health status ‘to the point where a medical or nursing review may be needed’ (O'Neill et al., 2018, p. e993), provides an opportunity to intervene early, thus preventing further/severe physiological derangement and subsequent adverse outcomes (Campbell et al., 2020). Detecting early warning signs of acute deterioration and implementing appropriate management plans in the residential aged care facilities (in‐house), are therefore essential to prevent further deterioration that can result in a transfer to hospital (Laging et al., 2018).

Early warning scoring systems (EWSSs) are tools that assign a numeric value to physiological parameters (e.g., heart rate) to derive a score and identify whether a person is deteriorating (Smith et al., 2014). These tools use the principles of the primary survey of airways (clear airway), breathing (respiratory rate and effort), circulation (pulse rate and blood pressure), disability (level of consciousness), and exposure (temperature and injury) and have been implemented internationally and in Australian tertiary care hospitals to improve the detection of acute deterioration (Australian Commission on Safety and Quality in Health Care, 2019). The impacts of using these tools include improvements in vital sign documentation, culminating in fewer unplanned intensive care unit admissions and hospital deaths (Mitchell et al., 2010). An example of an early warning scoring system commonly used in tertiary care hospitals is the modified early warning score (MEWS) (Downey et al., 2017), which is also used to predict the length of stay, in‐hospital outcomes, critical care admissions, and mortality.

However, early warning score systems rely on monitoring trends in clinical parameters (blood pressure, temperature, pulse, respiratory rate, and oxygen saturation) to be tracked and documented daily (Downey et al., 2017). Physiological changes that occur with aging alter the presentation of acute illness in the older person and changes in these clinical parameters may only occur once the disease is well advanced, if at all (Jarvis, 2016). Further, unlike tertiary care settings, residential aged care provides residents with a more home‐like environment with greater emphasis on supporting health, wellbeing, social life, and safety (Australian Government: My Aged Care, n.d.) and daily monitoring of clinical parameters is not routine care for all residents. Finally, the unique skill mix of the aged care workforce can be problematic as the majority of the workforce do not have the skills to conduct this level of assessment (Australian Royal Commission into Aged Care Quality and Safety, 2021; Boockvar et al., 2000). These factors are barriers to the acceptability, feasibility, and validity of using existing early warning score systems in residential aged care.

The aged care workforce comprises regulated (e.g., nurse practitioners, registered nurses, and enrolled nurses/licensed vocational nurses) and unregulated care staff (personal care workers/assistants; assistants in nursing), and other non‐clinical personnel (Australian College of Nursing, 2020). Between 2003 and 2016, the number of registered nurses (21%–14.4%) and enrolled nurses (14.4%–9.3%) declined and the number of personal care workers/assistants in nursing increased from 56.5% to over 70% of residential aged care staff (Eagar et al., 2019), a trend that is being seen internationally (OECDiLibrary, 2019). Only regulated nursing staff have adequate training to comprehensively assess for the signs and symptoms of deterioration (Eagar et al., 2019) and to use validated early warning assessment tools. However, unregulated staff, who spend most time with residents, are better positioned to detect the early warning signs of acute deterioration, though do not have training to detect variation in clinical parameters, including monitoring vital signs. This may arguably contribute to the failure to detect signs of deterioration which delays initiating appropriate action (Australian Royal Commission into Aged Care Quality and Safety, 2021). To inform the development of an acceptable and feasible early warning system for residential aged care and support for the aged care workforce in a timely manner, a rapid review of literature was conducted that aimed to identify the clinical indicators of acute deterioration in residents and to identify the factors that influence residential aged care facility staff's identification of these.

## METHODS

Due to the current crisis in aged care, as declared by the Australian Royal Commission into Aged Care Quality and Safety (2021), a rapid review was undertaken to expediate the process to allow findings to inform the other phases of work being done by authors; to develop an early warning tool specifically designed for use in residential aged care settings to detect the early clinical indicators of acute deterioration in residents. The rapid processes used for this review were underpinned by the WHO rapid review guidelines (Tricco et al., 2017) and the Cochrane Rapid Reviews Methods Group recommendations (Garritty et al., 2021). The review is reported against the Preferred Reporting Items for Systematic Reviews and Meta‐Analysis (PRIMSA) guidelines (Moher et al., 2009). The review protocol is registered with PROSPERO; registration number: CRD42021226996 (revised September 29, 2021). For the review purpose, it was assumed that nursing homes, care homes, skilled nursing facilities, long stay care homes, and special homes for institutionalized older persons are comparable to Australian residential aged care facilities.

### Search strategy

The review's eligibility criteria were formulated applying the mnemonic Condition, Context and Population (CoCoPop) system for review protocols (Joanna Briggs Institute, 2020). The condition of interest for this rapid review was to identify the clinical indicators that flag early deterioration of persons who reside in residential aged care settings (the context). The population of interest included persons who reside in residential aged care facilities and nursing and other care staff of such facilities.

Preliminary searches were undertaken in the CINAHL database using the relevant basic search terms “aged care”, “nursing home”, “residential aged care”, “deterioration”, clinical, and “early warning score”. The search strategy was then established with university health librarian assistance and the collective opinions of experts in the field. Search strings of keywords and index terms identified in the preliminary search results, together with relevant Medical Subject Heading (MeSH) terms, such as “Age Specific Care”, “Nursing Homes+”, “Residential Care+”, “Clinical Deterioration”, and “Early Warning Score” were used. This strategy was then tested in CINAHL and reviewed by peers (Garritty et al., 2021) and modified accordingly. The final search strategy was adapted slightly to suit the other databases searched (Medline, PubMed, and Cochrane Library). Searches were undertaken using the latest database and citation searching technologies (SC and MM) (Garritty et al., 2021). See Appendix S1 for search strategy examples for each database.

The reference lists of the accepted papers (upon full text review) were searched for additional studies by two authors (S. C. and A. S.).

### Eligibility criteria

Included articles were those reporting original research findings, relevant reviews (Garritty et al., 2021), and PhD or Masters Dissertations (only gray literature included) published between 2000 and January 2022 (to account for changes in technologies and aged care population trends). These publications reported outcomes related to the clinical indicators of acute deterioration of residents living in residential aged care settings, and factors that impact on and influence the identification of these. Excluded publications were those with populations not living or working in residential aged care settings, not published in English, single case studies or case reports, non‐peer reviewed papers, editorials, letters, discussion and expert opinion pieces, and other gray literature.

### Study selection

Records were de‐duplicated via automated processes in EndNote (X9.3.1). Two authors (S. C. and A. S.) independently screened all identified titles. A tailored abstract screening tool was developed (by S. C.) and piloted across 20 abstracts by two authors (S. C., A. S.), and was revised accordingly (Garritty et al., 2021). All remaining abstracts were dual screened independently by two authors (S. C. screened all abstracts and the other authors were allocated a selection of consecutive abstracts for the second review). The full‐text of selected records from the abstract screening process were independently reviewed by two authors (using the same methods as per abstract review) using a tailored full‐text review excel spreadsheet as a guide. Disagreements were resolved via discussion between the two reviewing authors, or with input from a third author as necessary.

### Data extraction

Relevant data were extracted from all included studies by S. C. and entered into the full‐text review excel spreadsheet. Other authors were each allocated a selection of publications to check extracted data for correctness and completeness (Garritty et al., 2021). To reach consensus, disagreements were discussed, and a third author consulted, as necessary (C. P. or M. M.). The data extracted included standard publication details, study purpose/aims, results relevant to this review (clinical indicators and factors that influence identification of these), details of interventions, and limitations.

### Quality assessment

Research publications were quality assessed using the 16‐item Quality Appraisal Tool in Studies with Diverse Designs (QATSDD) (Sirriyeh et al., 2012) on a 4‐point scale (item addressed: 0‐not at all; 1‐very slightly; 2‐moderately; 3‐completely). The critical appraisal skills programme (CASP) (Critical Appraisal Skills Programme, 2018) systematic review checklist was used to assess quality of review papers. The quality of the included publications was assessed by S. C. and a second author independently verified all judgments (and supporting statements) (Garritty et al., 2021). Percentage of inter‐rater agreements and an average quality score across all included records were computed (Sirriyeh et al., 2012). Publications were not excluded if deemed to be of poor quality, rather the collective quality of the included papers was of interest.

### Synthesis

Due to the heterogeneity of study designs, a narrative analysis was used to synthesize study findings. As the primary survey (Airways, Breathing, Circulation, Disability, Exposure [ABCDE]) underpins the data collected by early warning scoring systems and is the gold standard approach to assessing acute deterioration globally (Resuscitation Council UK, 2014), the identified clinical indicators were categorized by clinically trained authors (all but S. C.), in a series of workshops, using this framework as a means of identifying and organizing the most common clinical indicators used in practice. An ‘Other’ category captured indicators that did not fit within the Airways, Breathing, Circulation, Disability, Exposure categories, and were grouped logically. Factors that influence the identification of acute deterioration were categorized according to the source: consumers (residents and their families), workforce/others, and organization (Australian Government: Aged Care Quality and Safety Commission, 2021). Consensus of categories of factors was reached across all authors through a series of workshops.

## RESULTS

Of the 2127 records identified, 840 duplicates were removed, 1213 were excluded upon title and abstract screening and 74 papers were full text reviewed. Of these, 54 were excluded due to not meeting selection criteria; 20 records were included (19 research papers and one review paper). Reasons for exclusion were divided into six categories, the most common was not aligning to the review's aims (not relevant) (*n* = 43), and not being based in residential aged care facilities (wrong setting) (*n* = 8), e.g., hospital based. See full details in Figure S2.

### Quality assessment

Quality assessment interrater agreement of the 19 research publications and the review paper was 93% and 60% respectively. These 19 research publications were collectively rated to be less than moderate quality (median 1.7, range 0.8–2.5) from a possible maximum score of 3 (higher quality). The review paper (Laging et al., 2015) met six of the 10 CASP quality items (Critical Appraisal Skills Programme, 2018). Quality assessment scores can be found under author details in Tables 1 and 2.

**TABLE 1:** Matrix – clinical indicators of acute deterioration of residents of residential aged care facilities

Clinical indicators (quality score^a^)	Barker et al., 2020 (1.9)	Little et al., 2019 (1.8)	Ouslander et al., 2018 (0.9)	Stansfield, 2012 (2.4)	Ashcraft & Owen, 2014 (1.6)	Tingström et al., 2010 (2.0)	Ouslander et al. 2016a (1.1)	Ashcraft & Champion, 2012 (1.1)	Ouslander et al. 2016b (1.1)	Cohen‐Mansfield & Lipson, 2006 (1.3)	Unroe et al., 2018 (1.6)	Boockvar et al., 2000 (1.9)	Boockvar & Lachs, 2003 (1.7)	Stocker et al., 2021 (2.3)
Airway														
New/changed cough			✓								✓			
Dysphagia										✓				
Breathing														
Work of breathing		✓	✓		✓		✓	✓	✓	✓				
Altered vital signs	✓		✓		✓		✓	✓	✓		✓			✓
Circulation														
Altered vital signs	✓		✓		✓		✓	✓	✓	✓	✓			✓
Altered circulation			✓		✓			✓			✓			
Chest pain					✓			✓		✓				
Disability														
Altered LOC		✓	✓		✓	✓	✓	✓	✓	✓	✓		✓	
Seizure					✓									
Changed behavior & mood		✓		✓	✓	✓	✓		✓		✓	✓	✓	
Fatigue & lethargy					✓	✓		✓		✓			✓	
Pain		✓	✓	✓	✓	✓	✓	✓	✓		✓			
Exposure														
Infection						✓			✓	✓	✓		✓	
Altered skin integrity		✓		✓										
Injury & falls					✓		✓		✓	✓	✓		✓	
Other														
Intake & output		✓			✓	✓	✓			✓		✓	✓	✓
Change in weight										✓				
Nausea & vomiting								✓			✓			
Abnormal labs					✓				✓					
Self‐reported complaints												✓		
Functional decline				✓	✓		✓		✓			✓	✓	
Change in continence		✓		✓					✓		✓		✓	

Abbreviation: LOC, Level of Consciousness.

^a^ Quality Assessment Score: Quality assessment tool for studies with diverse designs (QATSDD) – maximum score = 3 (higher score = higher quality).

**TABLE 2:** Matrix ‐ factors that influence the identification of acute deterioration of residents of residential aged care facilities

Factors (quality score^a^)	Laging et al., 2018, ^b^ (2.5)	Laging et al., 2015, ^c^ (60%^d^)	Little et al., 2019, ^e^ (1.8)	O'Neill et al., 2017a, ^f^ (2.2)	O'Neill et al., 2018, ^g^ (0.8)	O'Neill et al., 2017b, ^h^ (1.8)	Longo et al., 2004, ^i^ (1.6)	Boockvar et al., 2000, ^j^ (1.9)	Stocker et al., 2021, ^k^ (2.3)
Consumer‐related factors									
Consumers' knowledge of resident							P		
Social pressures: Pressure on nurses to perform					P				
Undervalued: Lack confidence in nurses						N			
Workforce/others‐related factors									
Communication issues		N/P			N		N		P
Strength of relationships		N/P					N/P		
Confidence									P
Knowledge and skills		N	P	P			N		
Undervalued: by work peers; stigma of working in RACF						N			
Role/scope of practice	N			P	N/P				
Social pressures: Response influenced by perceived expectations of others					P				
Knowing the resident					P	P	P		
Teamwork				P					
Organization‐related factors									
Under resourced									
Care staff skill mix	N	N/P			N	N	N		
Managing workload	N			N/P	N	N	N	N	
Knowledge and skills				P		N/P	N/P		
Equipment				P	N/P	N/P			
Access to healthcare professionals/services		N			N/P		N		
RACF culture									
RACF philosophy	N				N				
Undervalued: RN scope of practice minimized	N								
RACF structure/policy									
Role/scope of practice	N	N							
Policy							N		
Variation in services for RACFs		N							
Collaborate/communicate					N/P		N		

Abbreviations: N, Negative factor; N/P, Can be Negative or Positive, depends on circumstances, e.g., positive after hospital avoidance type intervention; P, Positive factor; RACF, Residential Aged Care Facility; RN, Registered Nurse.

^a^ Quality Assessment Score: Quality assessment tool for studies with diverse designs (QATSDD) – maximum score = 3 (higher score = higher quality).

^b^ Context: Recognition and value of nursing assessment in nursing home setting.

^c^ Context: Synthesis of qualitative papers about nursing home staff's decision making to transfer resident to hospital.

^d^ Quality assessment using CASP percentage of agreement of quality items.

^e^ Context: Post introduction of hospital avoidance program (14–15 months) – examined nursing staff's perceptions of managing deteriorating residents.

^f^ Context: Pre/post hospital avoidance program implementation – study looking at factors that influence nursing staff's intentions with and without a hospital avoidance program. **Target** –residents of residential aged care facilities. ^a^Social pressure from residents and family ‐ Multiple regression analysis: this was the only significant predicator of staff intention toward the **target action** (the early detection of deterioration and providing subacute care): **Time 1** Standardized beta = 0.0, *p* < 0.05; **Time 2** Standardized beta = 0.8, <0.001 with nursing staff mostly strongly influenced by perceptions of residents, family members and colleagues.

^g^ Context: Question aimed to elicit common attitudes and beliefs about nursing staff management of the deteriorating resident using Theory of Planned Behavior Framework.

^h^ Context: Illness identification in acute infection.

^i^ Context: Nursing assistants' observation/assessment of nursing home residents ‐ early warning tool development and validation.

^j^ Context: Care home stakeholders, most care home senior care staff perspectives of National Early Warning Score (NEWS) implementation in COVID‐19 environment.

^k^ Context: Post quality improvement program – introduction of Significant 7 (early warning tool).

### Characteristics of included studies

Included publications were from the USA, Australia, and from four European countries and 11 of the 20 were published between 2016 and 2021. One included paper is a PhD dissertation (Stansfield, 2012) and one a systematic review (Laging et al., 2015). The findings of this rapid review are made up of evaluations of a range of hospital avoidance projects, warning tools, and minimum data sets within the context of care home settings. Full details of the included studies can be found in Tables S3.1 and S3.2.

### Clinical indicators of early deterioration of residents *(106 mentions across* *n = 14* *publications)*

No article specifically listed the clinical indicators of acute deterioration of residents. Clinical indicators were extrapolated from items within early warning tools (Barker et al., 2020; Boockvar et al., 2000; Little et al., 2019; Ouslander et al., 2016a, 2016b), and specific variables within a minimum dataset used to screen residents (Stansfield, 2012). Other sources included reasons for/signs and symptoms exhibited by residents pre‐hospital transfer (Ashcraft & Champion, 2012; Ashcraft & Owen, 2014; Cohen‐Mansfield & Lipson, 2006; Ouslander et al. 2016a, 2016b; Ouslander et al., 2018; Unroe et al., 2018), non‐specific symptoms of residents during acute illness episodes (Boockvar & Lachs, 2003), or what Stocker et al. (2021) refer to as ‘soft signs’ of deterioration, and from unregulated care staff's opinions of how resident deterioration is determined (Tingström et al., 2010). Details of the data sources used to inform clinical indicators can be found in Table S4.

The target users of the early warning tools and/or the participants of studies were residential aged care nurses generally (Barker et al., 2020; Boockvar & Lachs, 2003; Little et al., 2019; Stocker et al., 2021) and more specifically unregulated care staff (personal care workers/assistants; assistants in nursing) (Boockvar et al., 2000; Ouslander et al., 2016a; Ouslander et al., 2018; Tingström et al., 2010) and a variety of regulated nursing staff, such as enrolled nurses/licensed vocational nurses (Ashcraft & Champion, 2012; Ashcraft & Owen, 2014), registered nurses (Ashcraft & Owen, 2014; Unroe et al., 2018), and nurse practitioners (Ashcraft & Owen, 2014; Cohen‐Mansfield & Lipson, 2006; Stansfield, 2012; Unroe et al., 2018). Two papers focused on physicians' perspectives of the signs and symptoms residents exhibit at time of hospital transfer (Ashcraft & Owen, 2014; Cohen‐Mansfield & Lipson, 2006). One paper provided views of stakeholders including National Health Service staff relevant to residential aged care (Stocker et al., 2021). Table S3.1 provides details of the studies that inform clinical indicators.

#### Early warning tools used in included studies

Early warning tools reported in the included publications include a purpose‐specific illness warning instrument (Boockvar et al., 2000), from which the Stop and Watch tool used in the INTERACT program was adapted (Ouslander et al., 2016a, 2016b), the Significant 7 (Little et al., 2019), and the National Early Warning Score (NEWS) (Barker et al., 2020; Stocker et al., 2021). Boockvar et al. (2000) report some validation statistics for their early warning instrument, however, there is no mention of the validation of the Stop and Watch tool in the INTERACT studies (Ouslander, et al., 2016a, 2016b); this tool was only mentioned briefly; these tools are targeted for use by unregulated care staff. Table S4 provides more information about the early warning tools used in included studies.

Below is a summary of the priority indicators of clinical deterioration as grouped into the five categories of primary survey (Airway, Breathing, Circulation, Disability, Exposure) and ‘Other’. See Table 1 for a matrix of these indicators. Table S5 provides additional details.

#### Airway

This category has the least number of identified clinical indicators (3%; 3/106) and comprises two subcategories; *new/changed cough* (Ouslander et al., 2018; Unroe et al., 2018) and *dysphagia* (Cohen‐Mansfield & Lipson, 2006), which includes aspiration.

#### Breathing

Clinical indicators that fit within the ‘Breathing’ category (14%; 15/106), consist of *work of breathing* (includes shortness of breath) and *altered vital signs* (such as oxygen saturation, respiratory rate, hypoxia), which were identified across 10 publications (Ashcraft & Champion, 2012; Ashcraft & Owen, 2014; Barker et al., 2020; Cohen‐Mansfield & Lipson, 2006; Little et al., 2019; Ouslander et al., 2016a, 2016b; Ouslander et al., 2018; Stocker et al., 2021; Unroe et al., 2018).

#### Circulation

Circulation related clinical indicators (15%; 16/106) included three subcategories; *altered vital signs* (Ashcraft & Champion, 2012; Ashcraft & Owen, 2014; Barker et al., 2020; Cohen‐Mansfield & Lipson, 2006; Ouslander et al., 2016a, 2016b; Ouslander et al., 2018; Stocker et al., 2021; Unroe et al., 2018) (e.g., pulse, blood pressure, temperature), *altered circulation* (Ashcraft & Champion, 2012; Ashcraft & Owen, 2014; Ouslander et al., 2018; Unroe et al., 2018) (such as bleeding, edema, arrhythmia) and *chest pain* (including chest tightness or pressure) (Ashcraft & Champion, 2012; Ashcraft & Owen, 2014; Cohen‐Mansfield & Lipson, 2006).

#### Disability

The most prominent category identified (32%; 34/106) across all but two publications (Barker et al., 2020; Stocker et al., 2021), ‘Disability’, included five subcategories. The most commonly identified ‘Disability’ was *altered level of consciousness* (such as confusion, mental status change) (Ashcraft & Champion, 2012; Ashcraft & Owen, 2014; Boockvar & Lachs, 2003; Cohen‐Mansfield & Lipson, 2006; Little et al., 2019; Ouslander et al., 2016a, 2016b; Ouslander et al., 2018; Tingström et al., 2010; Unroe et al., 2018), closely followed by *changed behavior and mood* related indicators (such as delirium, agitation, greeting change) (Ashcraft & Owen, 2014; Boockvar et al., 2000; Boockvar & Lachs, 2003; Little et al., 2019; Ouslander et al., 2016a, 2016b; Stansfield, 2012; Tingström et al., 2010; Unroe et al., 2018), and *pain* (Ashcraft & Champion, 2012; Ashcraft & Owen, 2014; Little et al., 2019; Ouslander et al., 2018; Ouslander, Naharci, Engstrom, Shutes, Wolf, Alpert, et al., 2016; Ouslander, Naharci, Engstrom, Shutes, Wolf, Rojido, et al., 2016; Stansfield, 2012; Tingström et al., 2010; Unroe et al., 2018).

#### Exposure

Exposure related indicators (12%; 13/106) included *infection* (Boockvar & Lachs, 2003; Cohen‐Mansfield & Lipson, 2006; Ouslander et al., 2016b; Tingström et al., 2010; Unroe et al., 2018), *altered skin integrity* (Little et al., 2019; Stansfield, 2012) and *injury and falls* (Ashcraft & Owen, 2014; Boockvar & Lachs, 2003; Cohen‐Mansfield & Lipson, 2006; Ouslander et al., 2016a, 2016b; Unroe et al., 2018).

##### Other

This category (24%; 25/106), which encompasses clinical indicators that did not fit into the primary survey categories, were grouped into seven subcategories (see Table S5 for full details): *intake and output* (such as hydration, decreased food intake) (Ashcraft & Owen, 2014; Boockvar et al., 2000; Boockvar & Lachs, 2003; Cohen‐Mansfield & Lipson, 2006; Little et al., 2019; Ouslander et al., 2016a; Stocker et al., 2021; Tingström et al., 2010) was the most frequently found ‘Other’ clinical indicator followed closely by *functional decline* (such as assistance required and weakness) (Ashcraft & Owen, 2014; Boockvar et al., 2000; Boockvar & Lachs, 2003; Ouslander et al., 2016a, 2016b; Stansfield, 2012) and *change in continence* (including bloody stool, diarrhea, impaction) (Boockvar & Lachs, 2003; Little et al., 2019; Ouslander et al., 2016b; Stansfield, 2012; Unroe et al., 2018).

### Factors that influence the identification of acute deterioration *(52 mentions across n = 9 publications)*

Factors that influence the identification of acute deterioration were inferred from a variety of study types reporting problems experienced by residential aged care staff when determining acute deterioration (Laging et al., 2018), factors influencing the decision to transfer a resident to hospital (Laging et al., 2015), and outcomes of hospital avoidance interventions (Little et al., 2019; O'Neill et al., 2017a; O'Neill et al., 2018). In addition, reported perceptions of the deteriorating resident, from the perspective of personnel (O'Neill et al., 2017b), using or developing early warning systems for aged care (Stocker et al., 2021), illness identification in the acute infection context (Longo et al., 2004), and problems identified while developing and validating a standardized illness warning instrument informed factors (Boockvar et al., 2000).

Targeted participants of the studies (e.g., a variety of aged care staff, residents and their family members) that informed factors that influence the identification of early clinical deterioration can be found in Table 3.2.

Table 2 summarizes the factors that influence the identification of acute deterioration of residents as grouped into three categories based on the origin of the issue: consumer, workforce and others, and organization‐related factors.

#### Consumer‐related factors

The term ‘consumer’ refers to the residents and their families or significant others. Few factors that relate specifically to consumers were identified (6%; 3/52). Three subcategories within this grouping include *consumers' knowledge of the resident* which provides clues to the resident's deterioration (Longo et al., 2004), *social pressures* placed on care staff from the consumers to detect early deterioration (O'Neill et al., 2018), and residential aged care facility staff's perception of being *undervalued* when consumers rate their expertise less than that of hospital staff (O'Neill et al., 2017b).

#### Workforce/others‐related factors

The workforce included regulated and unregulated staff working in residential aged care. This grouping (38%; 20/52) comprises four subcategories. The most populated sub‐category was staff *confidence* which was related to staff's deficit of ‘knowledge and skills’ to identify and manage acute deterioration (Laging et al., 2015; Longo et al., 2004). Increased knowledge and skills, after participating in hospital avoidance type projects and training (Little et al., 2019; O'Neill et al., 2017a; Stocker et al., 2021), improved the likelihood of identifying acute deterioration. ‘Knowing the resident’ (Longo et al., 2004; O'Neill et al., 2018; O'Neill, Reid‐Searl, et al., 2017), which can aid the early identification of acute deterioration, was often expressed in relation to unregulated care staff due to their time spent with residents. Erosion of registered nurses' ‘role and scope of practice’ (Laging et al., 2018), ‘social pressures’ from work peers (O'Neill et al., 2018), and feeling ‘undervalued’ (O'Neill et al., 2017b), too, weaken care staff's confidence. Other subcategories in this grouping include s*trength of relationships* (between nurses and physicians) (Laging et al., 2015; Longo et al., 2004), *communication issues* between unregulated care staff and registered nurses (Laging et al., 2015; O'Neill et al., 2018), and between registered nurses and physicians (Longo et al., 2004), including the use of a common language by nursing home staff on use of the National Early Warning Score (Stocker et al., 2021), and *teamwork* between unregulated care staff and registered nurses, which improved upon participation in a hospital avoidance program (O'Neill et al., 2017a).

#### Organization‐related factors

Organization refers to the care service specifically and/or the overarching governance of care practices. This grouping contains more than half of all the factors identified (56%; 29/52) across three subcategories. Being *under resourced* was the most frequently mentioned factor that can impact on the identification of deterioration in residents, in particular, ‘care staff mix’ (Laging et al., 2015; Laging et al., 2018; Longo et al., 2004; O'Neill et al., 2017b; O'Neill et al., 2018) (ratio of regulated and unregulated care staff (Laging et al., 2015)) and ‘workload’ issues (Boockvar et al., 2000; Laging et al., 2018; Longo et al., 2004; O'Neill et al., 2017a, 2017b; O'Neill et al., 2018), and inadequate/inconsistent staffing (O'Neill et al., 2018). Factors related to *residential aged care facility culture* include ‘residential aged care facility philosophy’, which values organization regimes rather than addressing an individual resident's needs (Laging et al., 2018; O'Neill et al., 2018), and staff feeling *‘undervalued’* by the organization evidenced by devolved responsibility to unregulated care staff (Laging et al., 2018). *Residential aged care facility structure/policy*‐related factors include nursing staff's ‘role/scope of practice’ being undermined by residential aged care facility organizations (Laging et al., 2015) and upon delegation of duties to less educated/trained care staff (Laging et al., 2018). Lack of opportunities to ‘collaborate/communicate’ with peers and with outside healthcare services (O'Neill et al., 2018) was viewed as problematic, in particular the lack of opportunity to directly communicate with physicians (Longo et al., 2004).

## DISCUSSION

Due to growing concerns of an increased incidence of avoidable hospitalizations of residents (Australian Royal Commission into Aged Care Quality and Safety, 2021) and associated risks, the impetus to detect acute deterioration of residents early and to provide them with more appropriate management in‐house is growing (Carter et al., 2020) with further work pending (Hodge et al., 2021). The Australian Commission on Safety and Quality in Health Care (2019) states that “recognising deterioration relies on detecting, understanding and interpreting abnormal vital signs and other observations” (Intention of – Responding to Acute Deterioration Standard), therefore, it is imperative that all care staff of residential aged care facilities can accurately identify early indicators of clinical deterioration. However, physical and psychological changes that occur with old age and impact all systems means that for even a well older person, the use of changes in vital signs and other assessment data collected during a primary survey is not a reliable indicator of acute deterioration. The validity of this method is further compromised when the older person has one or more chronic illness/es, which is true of the majority of older people in residential aged care (Australian Institute of Health and Wellbeing, 2019).

This review reports the common clinical indicators of deterioration of residents, as extrapolated from various sources. Of particular interest, few studies reported clinical indicators that align with the *Airway* category (Cohen‐Mansfield & Lipson, 2006; Ouslander et al., 2018; Unroe et al., 2018), and *Breathing* or *Circulation* categories which suggests these indicators are either not commonly experienced by residents, not often assessed in this cohort, and/or not reported or researched as reasons for a transfer to acute care settings. Conversely, the many disability‐related clinical indicators, and those in the *Other* category, reflect important subtle changes that are frequently used in residential aged care as an indicator of acute deterioration. This was an interesting finding as these categories have few physiological indicators that are commonly used in the early warning scoring system but highlight several health status changes, such as the resident's mood and behavior (Ashcraft & Owen, 2014; Boockvar et al., 2000; Boockvar & Lachs, 2003; Little et al., 2019; Ouslander et al., 2016a, 2016b; Stansfield, 2012; Tingström et al., 2010; Unroe et al., 2018) and functional decline (Ashcraft & Owen, 2014; Boockvar et al., 2000; Boockvar & Lachs, 2003; Ouslander et al., 2016a, 2016b; Stansfield, 2012) which can be detected without specialized skills or equipment. Similarly, Ouslander et al. (2018) found that ‘most acute changes in skilled nursing facility residents’ conditions' were nonspecific and multiple, highlighting the multifactorial nature of acute changes in this cohort’ (p. 2259). Therefore, to circumvent unnecessary transfer of residents to acute care settings, the need to look not only for obvious physiological signs of deterioration in residents, such as altered vital signs, but also for subtle changes in residents' health status, demeanor, behavior and functioning, that may indicate early clinical deterioration, cannot be underestimated. However, to detect these observable changes in the resident, an understanding that such ‘soft’ signs could indicate the person is becoming acutely unwell (Stocker et al., 2021) and a familiarity with what the resident's usual state is, is necessary.

Some attempts have been made to develop and/or validate an early warning tool specifically to assist care staff of residential aged care facilities identify residents' acute clinical deterioration (Barker et al., 2020; Boockvar et al., 2000; Little et al., 2019). As part of the INTERACT program, the Stop and Watch tool has been used by certified nursing assistants since at least 2013 with some promising results, e.g., financial benefits and improved quality of life (Lee et al., 2016, p. 108). In opposition to the views expressed by Barker et al. (2020) that the National Early Warning Score is feasible, and useful (in the COVID‐19 context) (Stocker et al., 2021) in care home settings, the use of track and trigger tools to alert care staff of clinical deterioration in non‐acute settings has been criticized (Campbell et al., 2020; Hodge et al., 2019). Hodge et al. (2019) ‘query if the National Early Warning Score is the logical early warning tool to use in a resource constrained health and social care system’ (p. 5), particularly in light of the primary focus of the National Early Warning Score; hospital populations rather than frail nursing home residents. Similarly, others (Campbell et al., 2020) have aired concerns that track and trigger systems ‘have not been implemented with the same methodological rigor in non‐acute care setting as has been the case in acute care setting’ (p. 29). Even so, it is imperative that care staff are empowered to identify acute deterioration early in a consistent, structured, and evidence‐based manner, as residents with advanced deterioration will likely need more intense treatment and have a higher chance of being transferred to an acute care facility, which can result in poor outcomes (Fox et al., 2020).

Few studies provide more than a cursory mention of the initial identification of signs and symptoms related to acute deterioration that results in a hospital transfer, e.g., use of the Stop and Watch tool (Ouslander et al., 2016a), which possibly devalues this important first step in addressing such deterioration. In contrast, this review provides evidence to inform the development of an acute warning tool tailored to residential aged care settings to provide a consistent approach to the early identification of clinical deterioration by care staff, as clinical signs and symptoms that would normally trigger an early warning system alert in an acute care setting may not capture the subtle changes in condition of older and frail persons with multiple complex health conditions (Hodge et al., 2019). These findings also highlight the need to monitor the health status of all residents daily to identify changing trends in the clinical indicators. For this to occur, key barriers to the early detection of acute deterioration: workforce knowledge and skill deficits and inadequate resourcing (Australian Royal Commission into Aged Care Quality and Safety, 2021), should be addressed.

Most factors that influence the identification of clinical deterioration in residential aged care facilities were related to organizations being under resourced (Boockvar et al., 2000; Laging et al., 2015; Laging et al., 2018; Longo et al., 2004; O'Neill et al., 2017a, 2017b; O'Neill et al., 2018). There were minimal consumer‐related factors that assisted with the identification of the deteriorating resident. Of interest, feeling undervalued by consumers, workplace colleagues (O'Neill et al., 2017b) and the organization (Laging et al., 2018) hindered residential aged care facility staff's ability to identify the deteriorating resident. Some factors positively influenced the identification of acute deterioration, such as the implementation of hospital avoidance projects (Little et al., 2019; O'Neill et al., 2017a; O'Neill et al., 2018) and for some the implementation of the National Early Warning Score (Stocker et al., 2021) due to increased skills and confidence to work to their scope of practice and use of a common clinical language. Most factors however were reported negatively, such as communication failure (Longo et al., 2004; O'Neill et al., 2018), lack of access to outside health professionals (Laging et al., 2015), care staff skill mix issues (Laging et al., 2015), limited scope of practice to be able to provide the necessary care to residents (and as limited by the organization) (Laging et al., 2018), and workload problems (Boockvar et al., 2000; Laging et al., 2018; Longo et al., 2004; O'Neill et al., 2017a, 2017b; O'Neill et al., 2018). Future programs should consider these factors when developing resources to improve the detection of the early warning signs of deterioration in residential aged care facilities.

Staff confidence within the residential aged care facility workforce was a prominent issue related to identifying acute deterioration in residents. Unregulated care staff of residential aged facilities (personal care workers/assistants; assistants in nursing) spend more time with residents than regulated nursing staff (registered nurses; enrolled nurses), making unregulated staff pivotal to the early identification of acute clinical deterioration. However, the expertise of regulated nurses, too, is paramount to provide comprehensive clinical assessments and to enact interventions early (Australian College of Nursing, 2020). As such, both the regulated and unregulated care staff of residential aged care facilities should have access to setting specific early warning tools, and related training, to increase their confidence and to ensure residents receive appropriate and timely care as needed.

Limitations related to rapid review methodology, that increase the risk of bias, may impact the integrity of the findings due to less transparency and reproducibility, exclusion of gray literature (except one PhD thesis included), and due to the search beginning at the year 2000 which increases the risk of excluding significant results (Garritty et al., 2021). Furthermore, relevant studies may have been missed even though literature searches were undertaken using review‐specific strategies and due to the inclusion of English‐only publications; all included studies too were from developed countries. The variability of sample sizes and data sources, and the collated limitations of the included publications, further limit this review's findings, e.g., almost half of the studies were single site studies (Ashcraft & Champion, 2012; Boockvar et al., 2000; Boockvar & Lachs, 2003; Cohen‐Mansfield & Lipson, 2006; Little et al., 2019; O'Neill et al., 2017a, 2017b; O'Neill et al., 2018; Tingström et al., 2010). Clinical indicators sourced from health administrative data; hospital transfers (Ouslander et al., 2016a, 2016b), chart notes (Ashcraft & Champion, 2012), and discharge summaries (Unroe et al., 2018), were limited as these data were not collected, coded and recorded for research purposes, as such researchers had no control over data quality. The quality of the included publications, too, was low with the average scores across the original research publications equating to less than moderately meeting assessment criteria (Sirriyeh et al., 2012). While papers were not excluded due to being assessed as being of low quality, the quality assessment exercise demonstrates that high quality, rigorous studies are needed in this topic area. Due to the subjective nature of a narrative synthesis, others may interpret and synthesize the information extrapolated from included studies differently. Despite these limitations, this review provides the important first step toward developing an evidence‐based early warning tool specifically tailored to residential aged care facilities.

## CONCLUSIONS

This review highlights the complex nature of identifying the early signs of acute deterioration in residents and the need for validated tools with associated education and training to help build capacity of aged care nurses to manage acute deterioration in‐house. Subtle changes in residents' health status, demeanor, behavior, and functioning are used more frequently in residential aged care as early signs of acute deterioration and should be the focus of an early warning scoring system for residential aged care. In addition, education and support to build the capacity of the residential aged care workforce to identify and manage acute deterioration confidently and competently is recommended. The limited number of publications included in this review highlights the need for more well‐designed studies to build a robust body of knowledge that aims to increase the capacity of residential aged care facility care staff to identify acute deterioration early and to improve the implementation of proactive, targeted interventions to avoid hospitalization. In particular, the need for additional research in consumer‐focused factors, to ensure that identification of acute deterioration remains resident‐centric, is irrefutable. While the identification of clinical deterioration early in an illness trajectory may help avoid resident transfer to an acute care setting, and decrease pressure and expense on healthcare systems, it more importantly circumvents the resident experiencing stress and unnecessary exposure to iatrogenic illnesses (Ashcraft & Champion, 2012). Non‐regulated care staff, due to their more regular contact with residents, should be optimally trained to recognize and report the early signs of acute deterioration to promote the prompt response by regulated care staff who can enact appropriate interventions.

### CLINICAL RESOURCES

- WHO: Aging and Health https://www.who.int/news‐room/fact‐sheets/detail/ageing‐and‐health
- WHO: Integrated care for older people: guidelines on community‐level interventions to manage declines in intrinsic capacity https://www.who.int/publications/i/item/9789241550109

## CONFLICT OF INTEREST

There are no conflicts of interest to declare.

## Supporting information


Appendix S1

Figure S2

Table S3

Table S4

Table S5

## Data Availability

Data sharing is not applicable to this article as no new data were created or analyzed in this study.
