# Supplementary material for: Clinical indicators of acute deterioration in persons who reside in residential aged care facilities: A rapid review
Source: J Nurs Scholarsh. 2022 Oct 20;55(1):365–77. doi: 10.1111/jnu.12819 (PMC10092821; doi:10.1111/jnu.12819)
Supplement: Supplementary file 1 — Appendix S1 [file JNU-55-365-s003.docx]

**Supporting Information File 1: Example search strategy**

Please find below examples of database searches

**Search History: EBSCOhost CINAHL**

| **Search ID#** | **Search Terms** |
| --- | --- |
| S11 | S4 AND S10 |
| S10 | "early warning" OR "early detection" |
| S9 | (MH "Early Warning Score") |
| S8 | (MH "Early Warning Score") |
| S7 | S4 AND S6 |
| S6 | deterioration OR deteriorate OR deteriorating |
| S5 | S3 AND S4 |
| S4 | S1 OR S2 |
| S3 | (MH "Clinical Deterioration") |
| S2 | (MH "Nursing Homes+") |
| S1 | (MH "Age Specific Care") OR (MH "Residential Care+") |

**Search History: EBSCOhost Medline**

| [**Search ID#**](javascript:__doPostBack('ctl00$ctl00$FindField$FindField$historyControl$ReorderHistoryLink','')) | **Search Terms** |
| --- | --- |
| S8 | ""early warning score""  *(Limiters: Date 20000101-20201131; English; Human)* |
| S7 | S4 AND S6  *(Limiters: Date 20000101-20201131; English; Human)* |
| S6 | (MM "Clinical Deterioration") OR "deterioration OR deteriorate OR deteriorating" |
| S5 | S3 AND S4*(Limiters: Date: 20000101-20201131; English)* |
| S4 | S1 OR S2 |
| S3 | (MM "Clinical Deterioration") OR ""clinical deterioration"" |
| S2 | (MH "Nursing Home Patients") OR (MH "Nursing Homes+") OR ""nursing home*"" |
| S1 | (MM "Age Specific Care") OR (MM "Gerontologic Care") OR (MH "Residential Care+") OR (MH "Gerontologic Nursing+") |

| [**Search ID#**](javascript:__doPostBack('ctl00$ctl00$FindField$FindField$historyControl$ReorderHistoryLink','')) | **Search Terms** |
| --- | --- |
| S8 | ((MM "Clinical Deterioration") OR (MM "Cognitive Dysfunction") OR "deterioration OR deteriorate OR deteriorating") AND (S4 AND S7) *(Limiters: Date: 20000101-20201131; English)* |
| S7 | (MM "Clinical Deterioration") OR (MM "Cognitive Dysfunction") OR "deterioration OR deteriorate OR deteriorating" |
| S6 | S3 AND S4  *(Limiters: Date: 20000101-20201131; English; Human)* |
| S5 | S3 AND S4 |
| S4 | S1 OR S2 |
| S3 | (MM "Clinical Deterioration") OR ""Clinical deterioration"" |
| S2 | (MM "Nursing Homes+") OR ""nursing home*"" |
| S1 | ""age specific care" OR "Residential care"" |

**Search History: PubMed**

| **Search Term** |
| --- |
| ( "aged care" OR "nursing home" OR "residential aged care" OR "residential aged care facilit*" OR "long term care" ) AND ( deterioration OR deteriorate OR deteriorating ) AND clinical. Filters: from 2000/1/1 - 2020/12/31 |

**Search History: Cochrane database**

| **Search ID#** | **Search Terms** |
| --- | --- |
| #3 | #1 AND #2 |
| #2 | “aged care” OR “residential aged care” OR “long term care” |
| #1 | “clinical deterioration” |

| **Other Search Terms used** |
| --- |
| “clinical deterioration” and “aged care” |
| “early warning system*” |
| “early warning system*” AND “aged care” |
| “Residential aged care” OR “residential care” OR “nursing home” OR “nursing homes” OR “aged care home” OR “aged care” OR “residential care” OR residential OR “nursing facility” OR “long term care home” OR “long-term residential care” OR “long-term care facilities” OR “nursing home care” OR “care home” OR “residential facilities” OR “long-term care” OR “residential facilities” OR “assisted living facility” OR "care homes" OR "long term care" |
| Deterioration |
| “Clinical deterioration” |
| “Residential aged care” AND “early warning” |
| “Residential aged care” |
| "nursing home*" OR "long term care" OR "aged care" |
