# Supplementary material for: Clinical indicators of acute deterioration in persons who reside in residential aged care facilities: A rapid review
Source: J Nurs Scholarsh. 2022 Oct 20;55(1):365–77. doi: 10.1111/jnu.12819 (PMC10092821; doi:10.1111/jnu.12819)
Supplement: Supplementary file 2 — Figure S2 [file JNU-55-365-s002.docx]

**Supporting Information File 2: PRISMA Flow Diagram**

**RACF:** Residential Aged Care Facility

**^1^** Other methods - relevant publications identified from included publications’ reference lists.

**^2^** Some papers were rejected for more than one reason, hence total equates to more than number of papers excluded.

**Figure S2:** PRISMA Flow diagram outlining search results
