# Supplementary material for: Clinical indicators of acute deterioration in persons who reside in residential aged care facilities: A rapid review
Source: J Nurs Scholarsh. 2022 Oct 20;55(1):365–77. doi: 10.1111/jnu.12819 (PMC10092821; doi:10.1111/jnu.12819)
Supplement: Supplementary file 3 — Table S3 [file JNU-55-365-s004.docx]

**Supporting Information File 3: Summary Tables**

**Table S3.1:** Summary table – studies that inform clinical indicators of acute deterioration in residents of residential aged care facilities

| Author/Year  /Country/ Quality Assessment^1^ | Design/Aim | Sample/Setting | Findings relevant to Review |
| --- | --- | --- | --- |
| Barker et al  2020 England  1.9 | *Design:* Quantitative; Intervention evaluation  *Aim:* Explore feasibility of measuring and describing distribution of National Early Warning Score (NEWS) in NH | n=19,604 NEWS measures analyzed  Of n=2,424 residents (aged >65 years); Mean age 85 years  *Setting:* 46 NHs | NEWS feasible in the NH setting  Median NEWS=2. Two thirds (66%) of residents had a low NEWS (≤2), and 28% had a score of 0. 6,277 (32%) were routine readings and 2,256 (12%) were measured due to staff concerns.  Score distribution consistent with out-of-hospital settings |
| Little et al 2019  UK-England  1.8 | *Design:* Quantitative, Quality improvement (Intervention) study  *Aim:* Introduce Significant 7 (early warning tool) to identify and manage deterioration | n=9 Stakeholder group: n=1 Care home deputy manager; n=3 Senior carers; n=5 Carers  n=37 participants: n=15 Staff members trained; n=22 Residents  *Setting:* Residential unit in 87-bed NH | Primary Outcomes: Reduction in pressure ulcers from 8 (pre intervention) to 1 (post); Reduction in resident falls |
| Ouslander et al  2018^2^ USA  0.9 | *Design:* Quantitative, 2^nd^ Analysis of RCT data  *Aim:* Presentation and management of acute changes in condition in SNFs during implementation of Change in Condition (CIC) Tool to reduce unnecessary hospital visits | Data from n=133 SNFs (out of the n=264 SNF in the RCT; ~50%):  n=55 SNFs intervention; Mean age of residents (for which CIC tool without Transfer submitted) 80.6 years (SD 11.1)  n=78 SNFs control; Mean age of residents 80.8 years (SD 10.8) for which no CIC tool submitted  Analyzed 7,689 episodes of acute change in condition using the CIC tools  *Setting:* SNFs | Most of the 7,689 episodes of acute condition change reported involved multiple non-disease specific changes  OR for transfer > 2 for acute changes: mental status change, abnormal vital signs, bleeding, shortness of breath, unresponsiveness (highest OR 3.83)  Most reported changes in condition: function, mental status  Most reported new symptoms: pain, abnormal vital signs, cough  Most common abnormal diagnostic test results: X-ray, urinalysis or urine culture, complete blood count  Least likely associated with transfer: skin conditions and wounds; cough; urinary symptoms; abnormal urinalysis (OR <1) |
| Stansfield, 2012  USA  2.4 | *Design:* Quantitative, prospective, Ex Post Facto Quasi-Experimental  *Aims:* Assess efficacy of Nurse Practitioner Consultative Program (NPP) to promote care-in-place; and assess outcomes compared with transfer/return care | n=261 eligible incidents to assess  n=175 to assess H_1_ & H_2_, of n=143 residents (47 males; 96 females, aged 67-104 years, Mean age 84.19 years [SD 7.79])  n=86 to assess H_3_ & H_4,_ of n=70 residents (27 males, 43 females)  *Setting:* 8 regional NH (bed range: 39 to 248) where 5 NH incorporated NP assessment into practice, 2 use the services less frequently and 1 very seldom | Reported changes in conditions leading to hospital transfer: cardiac, catheter issue, dehydration, endocrine, gastrointestinal, infection, neurological, no information, pain, psychiatric, respiratory, trauma, vascular  H_1,2_ NPP result in more incidences of care-in-place and fewer incidences of transfer/return than usual care  H_3,4_ scores indicating deterioration in health and function would be fewer in the care-in-place groups than the transfer/return group |
| Ashcraft & Owen  2014  USA  1.6 | *Design:* Quantitative, Descriptive Survey Study  *Aims:* Identify signs and symptoms exhibited by NH residents at time of hospital transfer and strategies health care providers use to prevent transfer | n=108 NH health care providers: n=75 LVN (69%); n=27 RN (25%); n=2 NP (2%); n=1 PA (1%); n=3 Physicians (3%)  *Setting:* Random selection of 50 rural and 50 urban NH in Texas sent survey | Top 6 ranked (in importance and frequency order) signs and symptoms at time of transfer: SOB; Change in level of consciousness; Decreased oxygen; Chest pressure or tightness; Weakness/fatigue/lethargy; Muscle /or bone pain  10 signs and symptoms from open-ended questions (frequency order): Fall with injury, Pain, Abnormal labs, Abnormal vital signs, Abnormal bleeding, Seizure, behavior change, Cardiac arrest, Hydration, UTI |
| Tingström et al  2012  Sweden  2.0 | *Design:* Qualitative, cross sectional, part 1 of a prospective, longitudinal project  *Aim:* Explore early nonspecific signs and symptoms of infection in elderly institutionalised individuals described by NAs | n=21 NAs employed in 1 community care organization in southeast Sweden; Median age 50 years (range 22-61); All: female; Experience working in community care for elderly Median 18 years (range 4-34)  *Setting:* 2 special homes (urban community care organization) for the elderly in town of 15,000 population | 2 categories of non-specific signs and symptoms:  1. Is not as usual: described general discomfort related to possible infection, e.g., unrestrained behavior, aggressiveness, restlessness, confusion, tiredness and feebleness, and decreased eating  2. Seems to be ill: related to established infection in general terms of fever and pain or specifically related to pneumonia, UTI, skin infection, cold, eye infection |
| Ouslander, et al  2016a^2^  USA  1.1 | *Design:* Quantitative, RCT, Retrospective  *Aim:* Describe results of structured, retrospective RCA performed by SNF staff on hospital transfers to identify lessons for reducing transfers  *Note:* Intervention group received education, INTERACT resources and technical support to implement full INTERACT QI program. | n=4,856 Quality Improvement tools submitted during the 12 month implementation period  *Setting:* n=64 SNF (out of potential 88) of the n=71 SNFs randomized to immediate implementation group. Of the n=64 SNF: 56% for-profit; 13% rural; Average number of beds 139; Average proportion of short-stay (<100 days) residents 34%; Average licensed nurse (RN, LPN) hours per day 1.60; Average 5-star rating 3.52 | Most transfers precipitated by multiple symptoms and signs, many nonspeciﬁc  Common reasons for transfers (in >10% of transfers): Abnormal vital signs (33.4%); Altered mental status (27.9%); SOB (23.3%); Pain – uncontrolled (18.5%); Functional decline (15.6%); Behavioral symptoms (15.1%); Fever (12.1%); Decreased food or fluid intake (11.7%); Unresponsiveness (10.3%)  Most common combinations of reasons: Abnormal vital signs and SOB (9%); Fall and pain (7%); Altered mental status and behavioral symptoms (7%) |
| Ashcraft & Champion, 2012  USA  1.1 | *Design:* Quantitative, Retrospective, Descriptive  *Aim:* Describe NH resident symptomatology and medical diagnoses associated with NH to hospital transfers | Chart notes of n=101 residents who were transferred from NH to hospital for treatment. 71% Female. Majority of residents (n=84) admitted to the NH within previous 2 years  *Setting:* 1 Non-Profit, Urban, Continuing Care Retirement Center (combined NH/SNF) | Symptoms prior to transfer (frequency order): Fatigue, Lethargy or weakness (23%); SOB (20%); Change in level of consciousness (19%); Muscle or bone pain (19%); Elevated BP (17%); Decreased oxygenation (17%); Edema, swollen body part, Hematoma (17%); Chest pain, pressure or tightness (16%); Elevated heart rate (14%); Nausea or vomiting (14%); Decreased BP (13%); Elevated temperature (13%) |
| Ouslander, et al  2016b^2^ USA  1.1 | *Design*: Quantitative; RCT (of INTERACT quality improvement program)  *Aim*: Characteristics of SNF to hospital transfers that occur within 48 hours and 30 days of SNF admission based on RCAs performed by SNF staff. Identify areas for improving transitions between hospitals and SNFs | n=4,658 hospital transfers analyzed  *Setting:* n=64 SNF (of possible 88 SNF in group randomized to the intervention group) submitted hospital transfer information for RCA | Among those transferred within 48 hours or 30 days: More common – SOB; Less common - Falls, Functional decline, Suspected respiratory infection, and New urinary incontinence  Most common if transferred ≤ 48 hours: Abnormal vital signs; SOB, Altered mental status; Uncontrolled pain; Behavioral symptoms  More common when transferred <30 days: SOB  Significantly more common when transferred ≥30 days: Functional decline; Suspected respiratory infection; New onset of urinary incontinence  Most common abnormal test results associated with transfers within 48 hours and <30 days: Pulse oximetry and anemia - Anemia more common among those transferred <30 days after SNF admission versus ≥30 days after SNF admission |
| Cohen-Mansfield & Lipson, 2006 USA  1.3 | *Design*: Quantitative, Exploratory, hypothesis generating study  *Aim:* Processes and factors influencing Physicians' decision regarding NH resident hospitalization | n=7 Health Professionals (completed questionnaire): n=6 male RACF Physicians, n=1 female NP  Physicians reported consideration of hospitalization following a status change for n=52 residents.  *Note:* ~ 500 RACF residents were at risk of an event and of being sampled on any day  *Setting:* 1 x 14-Unit, Non-profit, 562-bed NH | Status change events that either lead (or not) to hospitalizations: Trouble breathing (*p*=.017); Aspiration/pneumonia; Fracture (*p*=.095); Hypotension; Heart trouble; Chest pain; Infection; Not eating/weight loss; Fall; Fever; Cardiovascular accident; Trouble swallowing; Lethargy; Loss of consciousness; Other  *Note:* Status change event - acute health status change |
| Unroe et al., 2018 USA  1.6 | *Design:* Quantitative, Retrospective (Part of OPTIMISTIC Project)  *Aim:* Relationship between NH resident risk conditions and signs and symptoms at time of acute transfer and diagnosis of conditions associated with potentially avoidable acute transfers | n=1,174 long stay residents: 66% aged ≥ 75 years, 62% female, ~50% transferred had moderate to severe cognitive impairment, 66% needed help with getting in and out of bed  These residents collectively experienced: 1,931 acute transfers during study period; n=54 hospital discharge status unknown; n=146 transfers originated outside the nursing facility (or details unknown).  *Setting:* 19 (USA) Indianapolis NHs that participated in the OPTIMISTIC project | Most common symptoms at time of (acute) transfer to ED/hospital: Behavioral or cognitive issues (31%); Falls, trauma or fracture (18%); Cardiovascular issues (17%); Respiratory issues (16.2%); Pain (11%); Infection or immune system problems (10%); GI symptoms (9%) |
| Boockvar et al  2000  USA  1.9 | *Design:* Quantitative, Observational, Cohort  *Aim:* Develop a validated, standardised instrument for communication with medical staff to enhance NAs' observation and documentation of acute illness signs | n=23 NAs (completed instrument on n=74 NH residents). NAs: 75% female; Median age 48 years; 48% completed high school; 13% had a college education; Median nursing experience 10 years; Median experience on current NH floor 2 years. Residents: 69% female; Median age 84 years (62-99); 60% White, 32% Black, 8% Hispanic; Median duration of residence 3.3 years (0-21); Median ADL Score 10 (5-12); 63% Dementia (MMSE score <20 and other)  *Setting:* 409-bed urban not-for-profit teaching NH affiliated with a large university medical center | Residents with instrument-recorded change more likely to develop acute illness within 7 days than those with no change (RR 4.1, 95% CI 2.6, 6.3).  NAs’ documentation of signs of illness preceded chart documentation by an average of 5 days.  Tool items include (*final tool items): Seemed like self; Weak*; Said hello or smiled (changed greeting)*; Nervous or agitated*; Self-reported complaints*; Ate the same amount of food*; Walked with a walker; Drowsy or tired; Confused; Needed help with dressing, toileting, or transfers; Self-reported health problems |
| Boockvar & Lachs  2003 USA  1.7 | *Design:* Quantitative, Prospective, Observational  *Aim:* Examine predictive value of nonspecific symptoms for acute illness in NH residents | n=202 (of 204 newly admitted) residents’ charts reviewed, of these: 69% female; Mean age 83 years (SD 8.7); 48% anticipated short NH stay; 57% disability in ≥4 ADL; 49% total dependence on others for locomotion; 38% dementia; 25% depressed mood  *Setting:* Academic 409-bed teaching NH in an urban setting | Falls were the most common non-specific symptom occurrence (in 7.1% in 10-day intervals).  PPV for acute illness were highest for: Lethargy (PPV 0.51)*; Weakness (PPV 0.50)*; Decreased appetite (PPV 0.46)*; Agitation and disorientation predicted an acute illness 1 in 3 times; Falls predicted an acute illness 1 in 4 times. |
| Stocker et al  2021  UK  2.3 | *Design:* Qualitative, Cross Sectional  *Aim:* To understand how a NEWS intervention has been used in care homes in one area of North-East England during COVID-19 | n=17 Stakeholders: n=10 Care Home Stakeholders (n=7 Managers; n=3 Care Home Senior Carers/Nurses) (NH Staff across 7 care homes (~30-50 beds) - of these 6 provide specialist dementia care and 1 of these also provided end of life care); n=7 NHS Staff (n=2 Directors, n=4 NHS Nurse Specialists in Older Persons’ Clinical Educators; n=1 Commissioning of the NHS service for older persons)   - *Setting:* 7 care homes in North-East England | If COVID-19 suspected, care home staff frequently  measured temperature and oxygen saturations as NEWS was not triggering a high level of concern. Stakeholders remained sceptical about sensitivity and specificity of NEWS clinical observations to identify COVID-19 in care home residents. |

**ADL:** Activities of Daily Living; **BP:** Blood Pressure; **CI:** Confidence Interval; **CIC:** Change in Condition (tool); **COVID-19**: Coronavirus 2019; **ED:** Emergency Department; **GI:** Gastrointestinal; **H_x_**: Hypothesis; **INTERACT** (program): Interventions to Reduce Acute Care Transfer; **LPN:** Licenced Practical Nurse**; LVN:** Licenced Vocational Nurse; **MMSE:** Mini Mental State Examination**; NA:** Nursing Assistant/s; **NEWS:** National Early Warning Score (tool); **NH:** Nursing Home/s; **OR:** Odds Ratio; **NHS:** National Health Service; **NP:** Nurse Practitioner**; NPP:** Nurse Practitioner (Consultative) Program; **OPTIMISTIC:** Optimizing Patient Transfers, Impacting Medical Quality, Improving Symptoms: Transforming Institutional Care; **PA:** Physician Assistant; **PPV:** Positive Predictive Values; **QI:** Quality Improvement; **RACF:** Residential Aged Care Facility; **RCA:** root cause analysis; **RCT:** Randomized Controlled Trial; **RN:** Registered Nurse; **RR**: Risk Ratio; **SD:** Standard Deviation; **SNF:** Skilled Nursing Facility; **SOB:** Shortness of Breath; **UK:** United Kingdom; **USA:** United States of America; **UTI:** Urinary Tract Infection

^1^ Quality Assessment Score: Quality assessment tool for studies with diverse designs (QATSDD) – maximum score=3 (higher score=higher quality)

^2^ These papers report results of the same study, noting that Ouslander et al. (2016b) is a secondary analysis of findings.

**Table S3.2:** Summary table - studies that inform the factors that influence the identification of acute deterioration of residents of residential age care facilities

| Author Year /Country /Quality Assessment^1^ | Design/Aim | Sample/Setting | Main Findings relevant to Review |
| --- | --- | --- | --- |
| Laging et al 2018  Australia  2.5 | *Design:* Qualitative, critical ethnography *Aim:* Explore the recognition and assessment of resident deterioration in the NH setting | n=66 participants: n=3 GPs; n=10 RNs; n=10 ENs; n=8 PCAs; n=20 Residents, n=15 Family members  *Setting:* 2 Australian NH:  NH 1: 30-bed; Ratio: RNs to Residents = 1:30; RNs supervising up to 5 ENs (no PCAs employed)  NH 2: 75-bed; Ratio: RNs to Residents = 1:73; RNs supervising up to 2 ENs and 11 PCAs | *Theme 1: Delegation and consistency of care:* Disengagement of RNs from residents; ENs and PCAs relied on to recognize and report deterioration  *Theme 2: Mass care with a task focus:* Staff focused on institutionalised regimes rather than individual need; Residents/family expected to know limits that reduce staff capacity; Organizational issues limit the time staff can focus on residents  *Theme 3: Undervaluing nursing assessment:* Erosion of role of RNs’ assessment; Undermining RNs’ clinical expertise; Task focused policies and organizational protocols; Devolved responsibilities to PCAs  *Theme 4: Perceiving NH roles:* Influence of NH philosophy of care on staff approach to assessment when resident deteriorates |
| Laging et al 2015  Australia  CASP 60% agreement | *Design*: Meta synthesis review of qualitative paper  *Aim:* Report a meta-synthesis of qualitative research studies exploring NH staff role in decisions to transfer residents to hospital | 17 qualitative papers included (Published 1989-2012). Includes perspectives of: RNs; ENs; NAs; DONs; NH Managers; LVNs; LPNs; Nurse Administrators; CNSs; NH Nurses; NH staff (undefined); Nurses  Of 14 studies which report sample sizes, range 7–60 participants, 3 studies did not report sample size  *Setting:* NH. Countries of included papers: USA x 7; Australia x 4; Canada x 4; Norway x 2 | *Theme 1: Lack of consensus of the NH role:* Debate among NH nurses regarding level of acute care that should be provided in-house  *Theme 2: Limited skills and confidence to manage resident on-site:* Poor staffing skill mix; NAs lack clinical acumen to recognize subtle symptoms; RNs lack confidence in their clinical skills and judgements; NAs frustrated by lack of nursing staff follow up  *Theme 3: Limited access to multidisciplinary services and resources:* RNs have limited scope to implement interventions without outside clinical support; Variation in medical services available to NHs; Cannot always access Drs, e.g., after hours  *Theme 4: Barriers to NH staff participation in the decision process:* No formal role for NH nurse in decision process; Inclusion in decision process relied on relationships and trust already established (with other health professionals and family)  *Theme 5: Challenges advocating on behalf of the resident:* Lack of clarified goals results care direction confusion; Resident’s versus family’s wishes; Families unrealistic expectations of transfer outcomes; Previous decisions can be overridden |
| Little et al 2019  UK-England  1.8 | *Design:* Quantitative, Quality improvement (Intervention) study  *Aim:* Introduce Significant 7 (early warning tool) to identify and manage deterioration | n=9 Stakeholder group: n=1 Care home deputy manager; n=3 Senior carers; n=5 Carers  n=37 participants: n=15 Staff members trained; n=22 Residents  *Setting:* Residential unit in 87-bed NH | *Primary Outcomes:* All staff members agreed or strongly agreed that the training had changed their practices for the better and 12 strongly agreed and 3 agreed that they felt confident that they could recognize deterioration |
| O’Neill et al 2017a^2^  Australia  2.2 | *Design:* Qualitative,  *Aim:* Examine NH nursing staff perceptions of their management of the deteriorating resident 14-15 months after hospital avoidance program introduced | n= 21 nursing staff: n=8 Nurses (RNs/ENs); n=13 NAs  *Setting:* 94-bed NH (where a pilot hospital avoidance program had been introduced) | *Theme 1: Valuing structure and support:* The program decision-support tools aided staff to assess and respond to resident deterioration  *Theme 2: Responding with confidence:* Program provided greater confidence in staff’s role in managing deteriorating resident  *Theme 3: Focusing on their role:* Program clearly defined and identified deteriorating health as an issue, which helped staff be more cognisant of their role in early recognition of, and response to, deterioration  *Theme 4: Working together:* Closer collaboration and teamwork of nursing staff since program (in particular, NAs working with nurses)  *Theme 5: Shifting the workload:* More work to care for deteriorating residents than to transfer them to hospital; Shift in time allocated to tasks and residents |
| O’Neill et al 2018^2^  Australia  0.8 | *Design:* Convergent parallel mixed methods (before-after hospital avoidance program) (Pragmatic theoretical approach) using TPB  *Aim:* Predict factors explaining NH staff intentions towards early detection of resident deterioration and providing care | n=75 nursing staff: n=15 RNs; n=10 ENs; n=50 AINs  Time 1 (T1): Pre-implementation of program: Mean age 49 years (SD 13); 91% female; 75% of sample completed T1 survey; 65% of sample in T1 focus group  Time 2 (T2): 14-15 months post implementation of program: Mean age 49 years (SD 14); 95% female; 49% of sample completed T2 survey; 28% of sample in T2 focus group  *Setting:* 94-bed NH | Behavioral intention of nursing staff towards the target behavior (detecting deterioration early/providing sub-acute care for residents) was determined by combined effect of:  *Attitude:* Nursing staff had a positive attitude towards target action at T1 and became significantly more positive at T2  *Subjective norm:* Social pressures felt from residents and family (the only significant predictor of staff intention towards the target action)  *Perceived behavioral control:* Staff members had a reasonable sense of control and confidence to carry out the target action; Main barriers identified towards the target action at both T1 and T2 were workload-related and revolved around prioritising routine, acute and subacute care and staffing |
| O’Neill et al 2017b  Australia  1.8 | *Design:* Qualitative, exploratory  *Aim:* Better understand aged care nursing staff perceptions regarding the deteriorating resident | n=49 nursing staff: n=12 RNs; n=7 ENs; n=30 PCWs  *Setting:* 94-bed NH | *Theme 1: Knowing the person:* Important to identify acute deterioration; care PCWs know residents intimately and are the first to notice changes  *Theme 2: Communicating changes:* Multiple stakeholders need to know of resident deterioration; Nurses central to communication process; Communication process is complex and time consuming while also caring for resident; Miscommunication (verbal and written) between RACF and hospital can occur during transfer  *Theme 3: Staying ‘home’:* NH staff believe hospitalization is traumatic, preferring residents remain in familiar surroundings; Access to diagnostic equipment and specialist training may mean better assessment allowing in-house care for resident  *Theme 4: What about me?* Other residents seek staff attention  *Theme 5: Workload implications:* Caring for a deteriorating resident adds to workload  *Theme 6: Feeling undervalued:* NH staff work not recognized by residents and other healthcare professionals for important work they do; Stigma of working in RACFs; knowledge skills and expertise not appreciated; Felt unappreciated by residents |
| Longo et al 2004  USA  1.6 | *Design:* Qualitative  *Note:* Study part of a larger project on lower respiratory infections in NH residents.  *Aim:* Investigate the process and timeliness of illness identification and initiating management for acute infection among NH residents | n=13 Professional staff involved in 6 episodes of acute illness, which include: n=7 Nurses (n=6 LPNs; n=1 RN; 1-22 years nursing experience; Worked in NH 0.25 years to 2 years); n=4 Physicians; n=2 Physicians’ Office Nurses  n=6 residents with acute illness, which include: Aged 74-95 years; n=3 male; n=3 female; Stayed in NH 2 months – 3 years; n=4 residents interviewed (n=2 not cognitively intact)  *Setting:* 4 NH | *Categories of factors affecting symptom recognition, illness identification:* Cross-Cutting Issues (e.g., workload issues, lack of staff training, work environment, timing); Symptom Recognition; Illness identification; Clinical Notification (e.g., relationships between nurses and physicians, miscommunication)  Communication was a crucial issue in identifying and managing acute illness episodes.  *6 Communication-related barriers:* Failure of communication medium; After hours illness onset with concomitant difficulty in contacting an on-call physician; Clinical decision-makers who interact through intermediaries; Communication of inappropriate or inaccurate information; Inadequate information transfer at shift changes; Prior relationship between staff nurse and physician  *Note:* Symptom recognition was also assisted by residents themselves, their family, or by other resident roommates – providing clues |
| Boockvar et al  2000  USA  1.9 | *Design:* Quantitative, Observational, Cohort  *Aim:* Develop a validated, standardised instrument for communication with medical staff to enhance NAs' observation and documentation of acute illness signs | n=23 NAs (completed instrument on n=74 NH residents): 75% female; Median age 48 years; 48% completed high school; 13% had a college education; Median nursing experience 10 years; Median experience on current NH floor 2 years  *Setting:* 409-bed urban not-for-profit teaching NH affiliated with a large university medical center | When NAs did not complete the early warning instrument (42% of work shifts) their most frequently cited reason was lack of time |
| Stocker et al  2021  UK  2.3 | *Design:* Qualitative, Cross Sectional  *Aim:* To understand how a NEWS intervention has been used in care homes in one area of North-East England during COVID-19 pandemic, and how it has influenced resident care | n=17 Stakeholders: n=10 Care Home Stakeholders (n=7 Managers; n=3 Care Home Senior Carers/Nurses) (NH Staff across 7 care homes (~30-50 beds) - of these 6 provide specialist dementia care and 1 of these also provided end of life care); n=7 NHS Staff (n=2 Directors, n=4 NHS Nurse Specialists in Older Persons’ Clinical Educators; n=1 Commissioning of the NHS service for older persons)   - *Setting:* 7 care homes in North-East England | With use of the NEWS in the context of COVID-19, care home stakeholders:  *Increased their confidence* to identify acute deterioration which backed up their intuition  *Used a common clinical language*, as per the NEWS assisted care home stakeholders to leverage for action when necessary |

**ADL:** Activities of Daily Living; **AIN:** Assistants in Nursing; **CASP:** Critical Appraisal Skills Programme, Systematic Review Checklist; **COVID-19:** Coronavirus 2019; **CNS:** Clinical Nurse Specialist; **DON:** Director of Nursing; **Drs:** Doctors**; EN:** Enrolled Nurse; **FG:** Focus Group; **GP:** General (Medicine) Practitioner**; LPN:** Licensed Practical Nurse; **LVN:** Licensed Vocational Nurse**; MMSE:** Mini-Mental Health Status Examination; **NEWS:** National Early Warning Score (tool); **NA:** Nursing Assistant**; NH:** Nursing home; **NHS:** National Health Service; **NP:** Nurse Practitioner; **PCA/W:** Personal Care Assistant/Worker; **RACF:** Residential Aged Care Facility; **RN:** Registered Nurse; **SNF:** Skilled Nursing Facilities; **T:** Time; **TPB:** Theory of Planned Behavior; **UK:** United Kingdom; **USA:** United States of America

^1^ Used Quality Assessment Tool for Studies with Diverse Designs (QATSDD) unless otherwise noted. QATSDD - maximum score=3 (higher score=higher quality); Used Critical Appraisal Skills Programme, systematic review checklist (CASP) to assess included review article. CASP - level of agreement across 10 quality questions (higher percent=higher quality)

^2^ These studies were undertaken as partial fulfillment of a PhD project
