# Supplementary material for: Clinical indicators of acute deterioration in persons who reside in residential aged care facilities: A rapid review
Source: J Nurs Scholarsh. 2022 Oct 20;55(1):365–77. doi: 10.1111/jnu.12819 (PMC10092821; doi:10.1111/jnu.12819)
Supplement: Supplementary file 4 — Table S4 [file JNU-55-365-s001.docx]

**Supporting Information File 4: Data sources used to inform clinical indicators of acute deterioration of residents who reside in residential aged care facilities**

**Table S4**: Data sources used to inform clinical indicators of acute deterioration of residents of residential aged care facilities

| Author Year  /Country  (Quality Score^1^) | Tool | Items Covered in Tool | Comments |
| --- | --- | --- | --- |
| Barker et al  2020  England  (1.9) | **NEWS** (National Early Warning Score) | - Pulse - Temperature - Systolic blood pressure - Oxygen saturation - Respiratory rate   *Responses to NEWS are based on levels of concern*, rated 0-3; lower numbers = less problems   - Low: 0-2 – Manage within the care home setting - Intermediate: 3-4 – Discussion with external healthcare professionals - High: 5-6 – Urgent response from external healthcare professionals - Critical: ≥7 – Emergency response, e.g., call ambulance | Explored feasibility of use of NEWS in NH. In this study, the NEWS appears to be targeted at RACF care staff generally. Barker and colleagues found the NEWS to be feasible for use in NH settings. Validation of this tool within this setting is pending. |
| Little et al  2019  UK-England  (1.8) | **Significant 7** (Early Warning Tool) | - Confusion - Mood - Pain - Hydration - Skin - Breathing - Toilet or bowel habits   *Response options:* Yes; No; and low, moderate or critical. | The Significant 7 tool provides guidance on how to manage signs and this tool (and training) was developed by the North East London Foundation Trust and Barking and Dagenham Havering Redbridge Clinical Commissioning groups. Tool validation not mentioned. Significant 7 appears to be targeted at informal carers but lacks an evidence base and validation. Tool seems to be specifically developed for use in RACFs. |
| Ouslander et al 2018  USA  (0.9) | **CIC** (Change in Condition without Transfer tool) | **General Change**   - Function - Mental status - Behavior - Skin or wound - Appetite - Pain level - Fluid level   **New Signs and Symptoms,** such as:   - Confusion or worsening cognition - Abnormal vital signs - Bleeding - Shortness of breath - Unresponsiveness - Pain - Cough - Urinary symptoms or incontinence - Fever - Behavioral symptoms - Nausea/vomiting - Falls   *Recorded as:* new or worsening  **Abnormal Test Results**   - X-ray - Urinalysis or urine culture - CBC - Pulse oximetry - Kidney function (BUN; Creatinine) - Blood sugars high - Other (such as Advance Care Directive, resident preferences or concerns, family preferences or concerns, clinician insisted on transfer despite staff willing to manage in facility, other   *Possible responses appear to be:* Yes or No | Study is an evaluation of a hospital avoidance intervention INTERACT program |
| Stansfield  2012  USA  (2.4) | **MDS 2.0 Cdn**  (Minimum Data Set Version 2, Canada) | Chosen clinical indicators from MDS 2 Cdn^2^  **Cognitive Patterns**   - Delirium   **Mood and Behavior Patterns**   - Mood - Behavior patterns - Alterability of behavior   **Physical Functioning and Structural Problems**   - Activities of daily living - Assistance required   **Continence**   - Bladder - Bowel   **Health Conditions**   - Pain frequency - Pain severity - Skin (count of ulcers by type)   *Possible responses*: Electronic - multiple dropdown boxes as possible responses – coded. | ‘MDS 2.0 Cdn is a comprehensive screening tool designed to capture resident’s health status, functional status, demographic profile and is specific to NH residents and is sensitive to change. *Note:* The MDS 2.0 Cdn is a mandatory assessment instrument used by all publicly funded NH in Canada – the assessment is conducted on admission, quarterly and in circumstances when the resident’s condition changes’ (p, 113). |
| Ashcraft & Owen  2014  USA  (1.6) | Purpose- developed data collection tool | **11 items**   - 7 items - demographic details - 4 items - directly related to aim of study, of which 2 questions are relevant to this review, which include: - Most common signs and symptoms that residents exhibit requiring transfer - Rank five most common signs and symptoms observed of residents from 11 item list | Aim of study was to identify common signs, symptoms and strategies used to prevent transfer to an acute care environment |
| Tingström et al 2010  Sweden  (2.0) | N/A Qualitative study |  | No specific tool mentioned, data sourced from focused interviews with nursing assistants |
| Ouslander et al 2016a  USA  (1.1) | **INTERACT tools** of particular interest **Stop and Watch** | - **S:** Seems different than usual - **T:** Talks or communicates less - **O:** Overall needs more help - **P:** Pain – new or worsening; Participated less in activities - **A:** Ate less - **N:** No bowel movement in 3 days; or diarrhea - **D:** Drank less - **W:** Weight change; swollen legs or feet - **A**: Agitated or nervous more than usual - **T:** Tired, weak, confused, or drowsy - **C:** Change in skin color or condition - **H:** Help with walking, transferring, toileting more than usual | Tool used by unlicenced/unregistered care staff member – these NH staff are to circle the change and notify a nurse.  *Note:* Situation, Background, Assessment, Recommendations (SBAR) approach also mentioned in paper – can be a verbal or written communication tool |
| Ashcraft & Champion  2012  USA  (1.1) | **Resident Characteristics Form** | Used Resident Characteristics Form to extract data (e.g., age, gender); symptoms retrieved at transfer by LVN, procedures performed prior to transfer, documented medical diagnoses from the day of NH admission and new medical diagnoses upon return to the NH collected. |  |
| Ouslander et al 2016b USA  (1.1) | **INTERACT QI tool** | Check boxes with specific items to facilitate summarising the data and space for narrative text. The tool has dichotomous (yes-no) questions at the end of a structured review used as a basis for determining preventability of hospital transfers. | Root Cause Analysis undertaken on data collected during an RCT of INTERACT quality improvement program. See INTERACT tools above (Ouslander et al., 2018; Ouslander et al., 2016a) |
| Cohen-Mansfield & Lipson  2006  USA  (1.3) | Study specific survey | **Questions on survey sought information about:**   - Status change of the NH resident - Decision-making process - Considerations in making treatment decisions - Role of advanced directives - Description of the status change event - Demographic information extracted from residents’ charts - Estimation of cognitive functioning of residents extracted from MDS-COGS (charts) | This survey was not a tool to assess residents’ acute deterioration as such. Rather, the description of the status change event was of interest for this review. |
| Unroe et al  2018  USA  (1.6) | **Transfer and Quality Improvement Form**  **Transition Visit Form** | - Documentation of known risk conditions - Description of signs and symptoms (from a predetermined dropdown list that the Center for Medicare and Medicaid Services [CMS] provides) - A rating of availability based on clinical judgement – as close as possible to the time of transfer - NPs complete the Transition Visit Form which includes hospital diagnoses, on return to facility. - Participant characteristic obtained from Minimum Data Set version 3 (MDS 3.0) assessments | OPTIMISTIC is a hospital avoidance type project - clinical model - multicomponent clinical intervention. This is about documentation after transfer has occurred.  OPTIMISTIC RNs were trained in INTERACT tools. When an OPTIMISTIC participant transferred, RNs performed a Root Cause Analysis. Upon transfer back, RNs and NPs collected data related to the ED visit or hospitalization. |
| Boockvar et al 2000  USA  (1.9) | **Illness Warning Instrument** (not specifically named) | **General question asked on how the resident is on the day**  ***Section 1:***   - Seemed like him/herself - Ate the same amount of food - Said “hello” or smiled at nurse^3^ - Watched TV between meals^3^ - Walked with a walker^3^   *Possible responses:* Yes today, the same as other days; No, not today different to other days  *Next questions:*   - Nervous or agitated - Drowsy or tired - Weak - Confused - Needed help with dressing, toileting or transfers   *Possible responses:* No, not today; or Yes today, but:  - The same as other days, or Worse than other days  ***Section 2:***   - Did resident or anyone else tell you he/she had a health problem today?   *Possible Reponses:* Yes; No – if yes, describe  **Final validated tool items**   - Weak - Said hello or smiled at you (change in greeting) - Nervous or agitated - Self-reported complaint - Ate the same amount of food - One or more of above | **Tool assessed:**   - Resident reported complaints - Global status change   Purpose developed instrument (Illness Warning Instrument) targeted for use by NAs. Pilot testing done to formulate instrument items, and to be easily comprehended by NA. This study also sought to validate the instrument.  Each instrument was unique to a particular resident, containing nine generic items and 0-3 patient-specific items specially formulated to be appropriate to the conduct of that resident. Patient specific items assessed change in greeting, activity and mobility ascertained in advance to be characteristic of the resident. |
| Boockvar & Lachs  2003  USA  (1.7) | No specific tool used | **Information collected via review of residents’ medical records include:**   - Demographics - Chronic comorbid conditions - Review of reported medical events that were possible episodes of acute illness - 5 ADLs (physical functioning metrics) | ADLs collected from NH MDS or from structured nursing notes (when the MDS was not available) and interviews with NH staff. |
| Stocker et al  2021  UK  (2.3) | N/A Qualitative Study |  | No specific tool mentioned, data sourced from semi-structured interviews with NH stakeholders (Senior care home carers and relevant NHS staff – healthcare professionals and commissioner) |

**ADL:** Activities of Daily Living; **BUN:** Blood Urea Nitrogen (test); **CBC:** Complete Blood Count (test); **ED:** Emergency Department; **INTERACT**: INTERvention to Reduce Acute Care Transfers; **LVN:** Licensed Vocational Nurse, **MDS:** Minimum Data Set; **MDS-COGS:** Minimum Data Set – Cognition Score; **NA:** Nurse Assistant; **NH:** Nursing Home/s; **NHS:** National Health Service; **NP:** Nurse Practitioner; **OPTIMISTIC**: Optimizing Patient Transfers, Impacting Medical Quality, Improving Symptoms: Transforming Institutional Care) **QI:** Quality Improvement; **RACF:** Residential Aged Care Facilities; **RCT:** Randomized Controlled Trial; **RN:** Registered Nurse

^1^ Quality Assessment Score: Quality assessment tool for studies with diverse designs (QATSDD) – maximum score=3 (higher score=higher quality)

^2^ MDS 2.0 Cdn variables chosen which have been associated with the Theory of Thriving with a change in thriving.

^3^ Tailored to specific resident.
