# Supplementary material for: Clinical indicators of acute deterioration in persons who reside in residential aged care facilities: A rapid review
Source: J Nurs Scholarsh. 2022 Oct 20;55(1):365–77. doi: 10.1111/jnu.12819 (PMC10092821; doi:10.1111/jnu.12819)
Supplement: Supplementary file 5 — Table S5 [file JNU-55-365-s005.docx]

**Supportive Information File 5: Extended matrix – Clinical Indicators of Acute Deterioration of residents of residential aged care facilities**

**Table S5:** Matrix – additional details of clinical indicators of acute deterioration of residents who reside in residential aged care facilities

| Clinical Indicators  (Quality Score^1^) | Barker et al 2020  (1.9) | Little et al  2019  (1.8) | Ouslander et al  2018  (0.9) | Stans-field  2012  (2.4) | Ashcraft & Owen  2014  (1.6) | Tingström et al  2010  (2.0) | Ouslander et al  2016a  (1.1) | Ashcraft & Champion  2012  (1.1) | Ouslander et al  2016b  (1.1) | Cohen-M & Lipson  2006  (1.3) | Unroe et al 2018  (1.6) | Boockvar et al 2000  (1.9) | Boockvar & Lachs  2003  (1.7) | Stocker et al  2021  (2.3) |
| --- | --- | --- | --- | --- | --- | --- | --- | --- | --- | --- | --- | --- | --- | --- |
| AIRWAY | | | | | | | | | | | | | |  |
| New/changed cough |  |  | ✓ |  |  |  |  |  |  |  | ✓ |  |  |  |
| Dysphagia – *aspiration, trouble swallowing* |  |  |  |  |  |  |  |  |  | ✓ |  |  |  |  |
| BREATHING | | | | | | | | | | | | | |  |
| Work of breathing – *shortness of breath, trouble breathing* |  | ✓ | ✓ |  | ✓ |  | ✓ | ✓ | ✓ | ✓ |  |  |  |  |
| Altered vital signs *– oxygen saturations, dec oxygen, respiratory rate, hypoxia* | ✓ |  | ✓ |  | ✓ |  | ✓ | ✓ | ✓ |  | ✓ |  |  | ✓ |
| CIRCULATION | | | | | | | | | | | | | |  |
| Altered vital signs – *pulse, temperature, fever, blood pressure* | ✓ |  | ✓ |  | ✓ |  | ✓ | ✓ | ✓ | ✓ | ✓ |  |  | ✓ |
| Altered circulation – *bleeding, cardiac arrest, edema, swollen, hematoma, arrhythmia* |  |  | ✓ |  | ✓ |  |  | ✓ |  |  | ✓ |  |  |  |
| Chest pain – *chest pressure, chest tightness* |  |  |  |  | ✓ |  |  | ✓ |  | ✓ |  |  |  |  |
| DISABILITY | | | | | | | | | | | | | |  |
| Altered LOC – *confusion, mental status change, unresponsive, worsening cog’ function, disorientation* |  | ✓ | ✓ |  | ✓ | ✓ | ✓ | ✓ | ✓ | ✓ | ✓ |  | ✓ |  |
| Seizure |  |  |  |  | ✓ |  |  |  |  |  |  |  |  |  |
| Changed behavior & mood – *Delirium, unrestrained behavior, aggressiveness, restlessness, depression, suicidal, greeting change, nervous, agitated* |  | ✓ |  | ✓ | ✓ | ✓ | ✓ |  | ✓ |  | ✓ | ✓ | ✓ |  |
| Fatigue & lethargy – *tiredness, feebleness* |  |  |  |  | ✓ | ✓ |  | ✓ |  | ✓ |  |  | ✓ |  |
| Pain – *muscle, bone, discomfort, uncontrolled* |  | ✓ | ✓ | ✓ | ✓ | ✓ | ✓ | ✓ | ✓ |  | ✓ |  |  |  |
| EXPOSURE | | | | | | | | | | | | | |  |
| Infection |  |  |  |  |  | ✓ |  |  | ✓ | ✓ | ✓ |  | ✓ |  |
| Altered skin integrity |  | ✓ |  | ✓ |  |  |  |  |  |  |  |  |  |  |
| Injury & falls |  |  |  |  | ✓ |  | ✓ |  | ✓ | ✓ | ✓ |  | ✓ |  |
| OTHER | | | | | | | | | | | | | |  |
| Intake & output – *hydration, decreased food, fluid & eating, appetite* |  | ✓ |  |  | ✓ | ✓ | ✓ |  |  | ✓ |  | ✓ | ✓ | ✓ |
| *Change in weight* |  |  |  |  |  |  |  |  |  | ✓ |  |  |  |  |
| Nausea & vomiting – *hematemesis* |  |  |  |  |  |  |  | ✓ |  |  | ✓ |  |  |  |
| Abnormal labs |  |  |  |  | ✓ |  |  |  | ✓ |  |  |  |  |  |
| Self-reported complaints |  |  |  |  |  |  |  |  |  |  |  | ✓ |  |  |
| Functional – *ADLs, assistance required, weakness* |  |  |  | ✓ | ✓ |  | ✓ |  | ✓ |  |  | ✓ | ✓ |  |
| Change in continence – *toilet/bowel habits, bladder, new incontinence,* *bloody stool, diarrhea, impaction* |  | ✓ |  | ✓ |  |  |  |  | ✓ |  | ✓ |  | ✓ |  |

**ADLs:** Activities of Daily Living; **Cog’**: cognitive; **LOS:** Level of Consciousness.

^1^ Quality Assessment Score: Quality assessment tool for studies with diverse designs (QATSDD) – maximum score=3 (higher score=higher quality)
